# Adipokines and Adipose Tissue: The Role and Use of Sodium-Glucose Co-Transporter-2 (SGLT-2) Inhibitors in Patients with Diabetes or Heart Failure

**DOI:** 10.3390/biomedicines13051098

**Published:** 2025-04-30

**Authors:** Michalina Mazurkiewicz, Patryk Bodnar, Dominika Blachut, Tomasz Chwalba, Wiktor Wagner, Eliza Barczyk, Ewa Romuk, Wojciech Jacheć, Celina Wojciechowska

**Affiliations:** 1 Second Department of Cardiology, Faculty of Medical Sciences in Zabrze, Medical University of Silesia, Specialistic Hospital in Zabrze, M. C. Skłodowskiej 10 Street, 41-800 Zabrze, Poland; dominikadyrcz@gmail.com (D.B.); tomaszarturchwalba@gmail.com (T.C.); wjachec@sum.edu.pl (W.J.); cwojciechowska@sum.edu.pl (C.W.); 2 Department of Anaesthesiology and Intensive Care, Clinical Hospital in Czeladź, Szpitalna 40 Street, 41-250 Czeladź, Poland; nuno38@interia.pl; 3 Student Research Team at the Second Department of Cardiology, Faculty of Medical Sciences in Zabrze, Medical University of Silesia, M. C. Skłodowskiej 10 Street, 41-800 Zabrze, Poland; wiktorwagner480@gmail.com (W.W.); eliza.barczyk@onet.pl (E.B.); 4 Department of Biochemistry, Faculty of Medical Sciences in Zabrze, Medical University of Silesia, Jordana 19 Street, 41-808 Zabrze, Poland; eromuk@sum.edu.pl

**Keywords:** adipose tissue, adipokines, adiponectin, heart failure, SGLT-2 inhibitors, flozins

## Abstract

Sodium-glucose co-transporter-2 (SGLT-2) inhibitors have become integral in treating both diabetes mellitus and heart failure, independent of left ventricular ejection fraction. Their pleiotropic effect influences multiple mechanisms, enhancing the function of various systems within the body. They exhibit nephroprotective and cardioprotective effects by improving cell metabolism, endothelial function, and slowing the fibrosis of the cardiac muscle, and they also have a beneficial impact on other organs. At the cellular level, they protect against the harmful effects of free radicals both by lowering glucose levels and by supporting the function of the antioxidant system. Moreover, SGLT-2 inhibitors can modify the metabolism of adipocytes by affecting the production of cytokines such as adiponectin—which increases insulin sensitivity, leading to weight loss and improved glycemic control.

## 1. Introduction

SGLT-2 inhibitors are a relatively new group of drugs that have found applications not only in diabetes but also in heart failure. A simple effect of their action is a reduction in glucose concentration, which may affect the body’s metabolic pathways.

Glucose metabolism abnormalities—including various forms of diabetes, insulin resistance, relative insulin deficiency, and progressive pancreatic β-cell dysfunction—are, at least in part, attributable to the dysregulation of adipokine secretion by adipose tissue [1].

The aim of this work is to summarize the current knowledge regarding the effects of SGLT-2 inhibitors on adipose tissue, including its metabolism as well as its autocrine and paracrine activity. Additionally, this review seeks to address whether, and by what mechanisms, the use of flozins influences body weight in patients with and without diabetes. Furthermore, it explores the impact of flozins on the production and function of adipokines, particularly adiponectin and leptin.

Databases such as Pubmed, Embase, Scopus, Google Scholar and Web of science were systematically searched without limitations in languages, mainly between 2018 and 2024. Additional articles were extracted from the reference lists of the retrieved articles, reviews, and meta-analysis on the topic. Specific terms like “SGLT-2 inhibitors”, “SGLT2i“, “flozins”, “adipocyte”, “adipokines”, “adiponectin”, “leptin”, “ghrelin”, “resistin”, “apelin”, “adipose tissue”, “weight loss”, ”diabetes mellitus type 2”, “heart failure” were selected to be the key words to sift out studies, which might be potentially relevant.

## 2. Adipocytes, Adipose Tissue, Gross Inspection, and Biological Role

Adipocytes, also called lipocytes and fat cells, are cells that mainly make up adipose tissue and specialize in storing energy in the form of fat. We distinguish between white adipose tissue (WAT) and brown adipose tissue (BAT). They consist of different types of fat cells. White adipose tissue (WAT) is the main source of energy in higher eukaryotes, and its primary purpose is to store triacylglycerols in periods of energy excess and to mobilize them in the event of energy deficiency [2].

These are plastic cells that can change their size and number in response to metabolism (adipogenesis or necrosis, apoptosis). Increased nutrition causes adipocytes to grow in size to store energy as adipose tissue, while starvation can cause adipose tissue to shrink [3].

**Figure 1:**
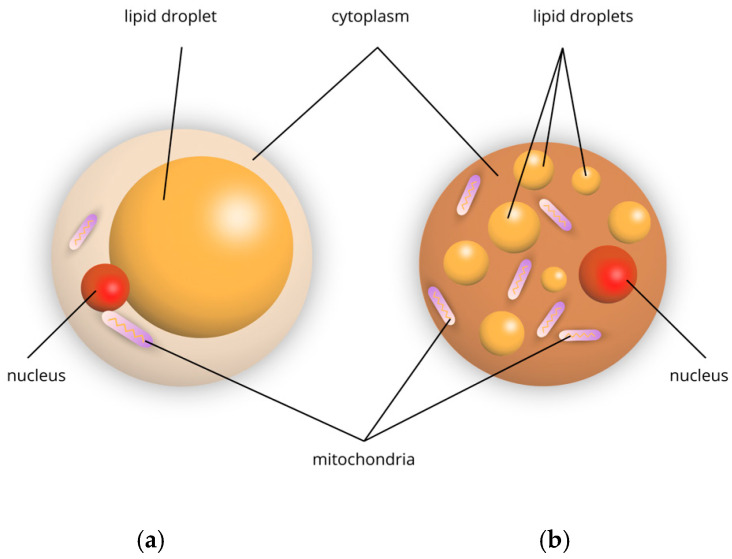
Schematic representation of adipocytes: (**a**) white, (**b**) brown.

The differences in structure and function between white and brown adipose tissue are as follows. White adipocytes are large round cells that contain one large lipid droplet and a thin cytoplasm that contains a peripheral nucleus. They store triacylglycerols as the main source of energy for the entire body. In addition, these cells exhibit endocrine functions, including the secretion of leptin, adiponectin, and pro-inflammatory cytokines [4]. Brown adipocytes are small cells consisting of several small lipid droplets and many mitochondria in their cytoplasm (Figure 1). They are responsible for regulating thermogenesis.

In this aspect of this review, we focus mainly on white adipose tissue (WAT) with attention to its metabolic effect. WAT is uniquely capable of storing large amounts of excess nutrients in the form of lipids. In turn, the accumulation of excess lipids in other tissues leads to insulin resistance [5]. Therefore, the proper breakdown of excess nutrients into WAT for storage or thermogenic fat for heat generation promotes metabolic health. It is worth noting that the site of adipose tissue expansion (into visceral or subcutaneous tissue) and the mechanism of expansion, through an increase in the number (hyperplasia) or size (hypertrophy) of adipose tissue, have a huge impact on metabolic health.

Adipose tissue proliferates through adipocyte hypertrophy (an increase in the size of fat cells) and/or hyperplasia (an increase in the number of fat cells). Hypertrophic growth is associated with higher levels of adipose tissue inflammation, fibrosis and hypoxia, as well as poor metabolic health [6]. In contrast, hyperplastic growth does not induce these pathological changes and is generally more metabolically favorable. The remodeling of adipose tissue induced by excess calories—which leads to obesity—involves dynamic and complex changes in the cellular composition of adipose tissue.

It is not the sheer number or size of adipocytes, but their ability to function properly hormonally and metabolically that determines how adipose tissue will affect glucose management. The review by Richard A.J. et al. addressed, among other things, the adipose tissue expandability hypothesis. If adipose tissue is unable to adequately increase cell number (hyperplasia) and/or cell size (hypertrophy) in a healthy manner, excess energy begins to be deposited in other organs (skeletal muscle, liver). Ectopic lipid accumulation contributes to the development of insulin resistance, which interferes with glucose metabolism (by, among other things, impaired muscle ability to uptake glucose and increased glucose production in the liver). Excessive or chronic inflammatory response in adipose tissue due to excessive cell size and uncontrolled cell death leads to impaired insulin signaling [7].

Consequently, a number of metabolic abnormalities, oxidative stress, mitochondrial dysfunction, immune dysfunction and chronic inflammation have been identified in the obese body [8,9]. When adipocytes reach storage capacity, cell death occurs, leading to activation of inflammation and fibrosis. Subsequent decline in WAT function leads to deleterious lipid accumulation in non-fatty organs (similar to lipodystrophy) [6]. An important issue is to determine whether defects in adipocyte differentiation lead to pathological adipose tissue remodeling, or whether they are a consequence of fibrosis, immune cell activity, or any of the many metabolic changes that occur with the metabolic syndrome. Subcutaneous adipocyte hyperplasia and healthy adipose tissue remodeling appear metabolically beneficial and contribute to systemic insulin sensitivity.

## 3. Effect of Glucose Concentration on Adipose Tissue Metabolism

A group of glucose transporters (GLUTs) that facilitate glucose absorption and metabolism in liver tissue, skeletal muscle, and adipose tissue play a very important role in ensuring the homeostatic control of blood glucose levels. Reduced glucose transport activity results in abnormal utilization of energy substrates and is associated with insulin resistance and type 2 diabetes. It is well known that GLUT4 is one of the key factors in whole-body glycemic control. However, the molecular mechanism of how insulin controls glucose transport across membranes and its relationship to impaired glycemic control in type 2 diabetes remains poorly understood. A number of circulating metabolites and hormone-like molecules, as well as potential additional glucose transporters, play a role in fine-tuning the flow of glucose between different organs in response to altered energy demands [10].

A review by Smith et al. [11] showed that a reduction in GLUT4, a major insulin-regulated glucose transporter, impairs lipogenesis in adipocytes synthesizing a new family of lipids secreted by adipose tissue (and potentially other tissues) and fatty acid hydroxy fatty acid esters (FAHFAs). FAHFAS have beneficial metabolic effects, including enhancing glucose uptake in adipocytes, increasing insulin-stimulated glucose transport and glucose-stimulated GLP1 secretion in the intestine and insulin secretion in pancreatic cells, and having potent anti-inflammatory effects. They cited that in an animal study, just a 3-day oral treatment of FAHFA reduced adipose tissue inflammation in obese mice. They further identified isomers of one FAHFA family, palmitic-acid-hydroxy fatty acid esters (PAHSA)-levels in serum and adipose tissue are low in insulin-resistant compared to insulin-sensitive individuals.

The interesting concept, “hypertrophic obesity”, in people at high risk of developing type 2 diabetes, is associated with insufficient numbers of new fat cells, leading to the enlargement of existing adipocytes (relative to their BMI), insulin resistance, and inflammation [12].

Elevated glucose levels promote glucose uptake through adipocytes and increased lipogenesis, leading to increased storage of triglycerides in fat cells. In turn, low glucose concentrations can promote the activation of lipolysis (through reduced insulin levels and an increase in hormones such as glucagon and catecholamines), resulting in the release of free fatty acids into the bloodstream. As a result, the balance between glucose levels, insulin levels and the activity of other hormones determines whether adipose tissue will mainly accumulate or release lipids [13,14].

## 4. Adipokines

Adipokines are a broad group of proteins (hormones, cytokines) secreted by adipose tissue. The novel function of adipose tissue was found in 1994 with discovery of leptin, the first member of adipokines. More than 600 adipokines have been identified, including the following: adiponectin, monocyte chemoattractant protein (MCP)-1, resistin, IL-1β interleukin (IL)-6, tumor necrosis factor (TNF)-α, omentin, ghrelin, and vaspin. It is expected to find many more adipokines secreted by adipose tissue, with apelin, fibroblast growth factor (FGF)-21 and neuregulin-4 being relatively “fresh” findings [15].

**Figure 2:**
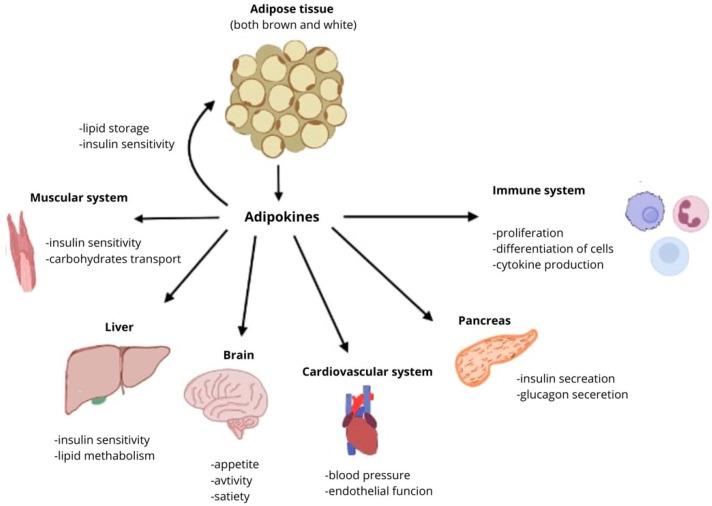
Effects of adipokines on different organs and systems.

Adipose tissue, especially white adipose tissue, is involved in the secretion of adipokines. These hormones have enormous influence on numerous systems in the human body, including cardiovascular system, nervous system, skeletal system, muscle system, gasto-intestinal system, immune system, neuroendocrine system, and the adipose tissue itself (Figure 2). Disorders in the secretion of adipokines can result in development and progression of various diseases: obesity, cardiovascular diseases (heart failure, atherosclerosis, coronary disease), autoimmune diseases (rheumatoid arthritis, systemic lupus erythematosus), diabetes, and even cancer (including liver, pancreatic, endometrial, colorectal, and post-menopausal breast cancer) [16,17,18].

The most important of these is adiponectin, as it exhibits anti-inflammatory and insulin-sensitizing effects (increases tissue sensitivity to insulin). Its concentration decreases with increasing body weight (especially in visceral obesity). Higher levels of adiponectin are associated with a lower risk of insulin resistance, type 2 diabetes and cardiovascular complications [19]. Below, there are detailed descriptions of the most important and well-known adipokines as well as two newly found ones.

### 4.1. Leptin

Discovered in 1994, leptin is an adipocyte-derived hormone, is 16 kDa in size, and is a combination of 167 amino acids. It presents a metabolic effect through LEP-R receptors [20]. The main role of leptin is the regulation of appetite and metabolism by inhibiting secretion of neuropeptide Y (which stimulates food intake) [21]. Leptin secretion is mainly concentrated in yellow adipose tissue; however, other tissues can also contribute, such as: brown adipose tissue (BAT), muscles, bone marrow, placenta, fetal tissue, teeth, and brain and stomach [22]. Leptin circulates in both free and protein-bonded form, with the first one being the active form [23]. Leptin metabolism disturbances are one of the factors resulting in developing obesity, including leptin resistance, leptin deficiency or secretion of damaged molecules [24]. Novel studies highlights leptin’s role in development of various illness: diabetes, cardiovascular: cardiac diseases, diabetic cardiomyopathy, cardiac fibrosis, vascular dysfunction, and skin diseases: psoriasis, systemic lupus erythematosus, and hidradenitis suppurativa [25,26,27].

### 4.2. Adiponectin

Discovered one year after leptin, adiponectin is a fat-derived hormone combined of 247-amino acid polypeptide [28]. There are two receptors for adiponectin: AdipoR1 and AdipoR2, which both can be located on skeletal muscles and liver [29].

Adiponectin improves insulin sensitivity by binding to AdipoR1 and AdipoR2, resulting in activation of the AMPK pathway [30]. AMPK then accelerates cellular metabolism and stimulates glucose uptake and fatty acid oxidation, thereby improving insulin sensitivity [31]. Moreover, it suppresses glucose production, suppresses gluconeogenesis and stimulates glucose assimilation independent of insulin levels [32].

In addition, it protects against atherosclerosis by suppressing the expression of monocyte adhesion molecules and the synthesis of inflammatory factors through inhibition of nuclear factor-kappa B [33,34]. It is also known that adiponectin targets extracellular signal-regulated kinase and thus inhibits vascular smooth muscle cell proliferation [35].

**Figure 3:**
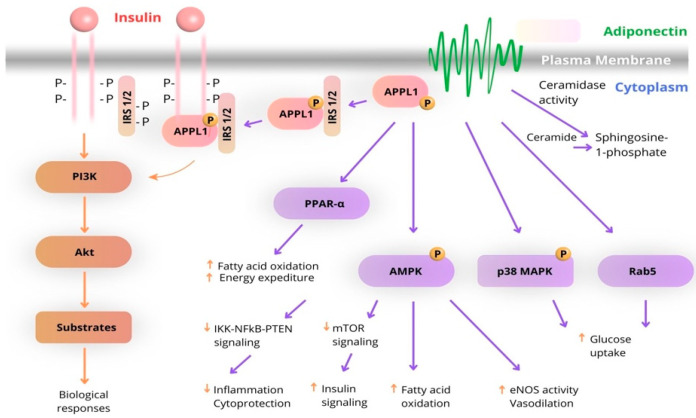
Schematic diagram of the interaction of adiponectin and insulin signaling pathways. (The arrows highlight the functional interplay between insulin and adiponectin signaling pathways).

Most of the metabolic effects of insulin are mediated by the PI3K/AKT pathway, leading to biological responses that include increased protein synthesis, lipogenesis, glucose uptake and utilization, and glycogen synthesis, as well as decreased lipolysis and gluconeogenesis. In the case of adiponectin, APPL1 interacts with AdipoR1 or AdipoR2 through its C-terminal PTB and CC domains and mediates the effects of adiponectin on the activation of multiple pathways, including PPAR-a, AMPK, and p38 MAPK. Both AdipoR1 and AdipoR2 are associated with ceramidase activity, which is activated upon adiponectin binding. One of the key binding partners of IRS1/2 is APPL1, which promotes the binding of IRS1/2 to the insulin receptor and enhances insulin signal transduction. This interaction between the insulin and adiponectin signaling pathways is the main mechanism by which adiponectin sensitizes insulin action in insulin target tissues (Figure 3) [36].

Its exact role in the pathophysiology of diseases is not completely investigated, However, certain disorders of its metabolism may be a factor in the development of cardiovascular diseases related to glucose and lipid metabolism, autoimmune diseases (such as rheumatoid arthritis, systemic lupus erythematous, and various myopathies) [37,38,39,40].

**Figure 4:**
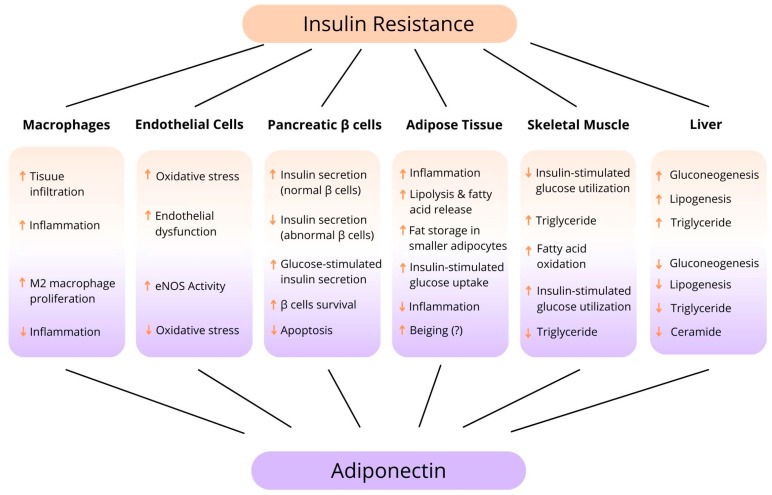
Tissue-specific function of adiponectin. (↑—increase, ↓—reduce).

In addition, we present a diagram of adiponectin’s action in relation to pathologies occurring in various tissues and organs that contribute to insulin resistance (Figure 4).

### 4.3. Resistin

A few years later, another adipokine was discovered: resistin. It is a small, circulating, 12.5 kDa cysteine-rich secretory protein that consists of 108 amino acids, secreted by adipose tissue [41]. It can mainly be found in peripheral blood mononuclear cells, macrophages, and bone marrow cells [42]. Circulating resistin was also observed in the pituitary gland, hypothalamus, epithelial cells from the gastrointestinal tract, pancreas, spleen, and skeletal muscle. Unlike previously described adipokines, there are no known resistin receptors; however, toll-like receptor 4 was reported as a potential one [43]. Metabolic effects of resistin are also very different from other adipokines. Resistin is a pro-inflammatory molecule, stimulating the transcription of pro-inflammatory genes, cytokines, and chemokines through NF-κB pathway causing endothelial dysfunction [44]. It contributes to the development of various diseases: obesity, diabetes (due to insulin resistance), ischemic heart disease, artherosclerosis, asthma, Leśniowski–Crohn disease, renal function impairment, osteoporosis, rheumatic disease, psoriasis, and even diverse types of cancer: breast, colorectal, liver, and lung [44,45,46].

### 4.4. Ghrelin

Ghrelin is a 28-amino acid acylated peptide with growth hormone secretagogue receptor (GHS-R) as its receptor, expressed widely in the anterior pituitary, thyroid gland, heart, pancreatic islets and various regions of the brain [47]. Similarly to other adipokines, it is secreted by adipose tissue. Its most known function is stimulation of the appetite, due to activation of hypothalamic neurocircuits [48]. The axis between leptin and ghrelin could be referred to as a kind of “ying-yang” in maintaining balance of food intake and its inhibition. A number of studies also established that ghrelin lowers blood glucose level and improves glucose tolerance [49]. Other remarkable roles of this adipokine are its cardioprotection and involvement in bone homeostasis [50,51]. Diseases in which ghrelin metabolism distributions are present are mainly metabolic diseases: obesity and diabetes and cardiovascular diseases: heart failure, fatal arrhythmias, myocardial infarction, and pulmonary hypertension [52,53].

### 4.5. Lipocalin-2

Lipocalin-2 (LCN-2) is a 198 amino acid adipocytokine, also referred to as siderocalin, neutrophil gelatinase-associated lipocalin (NGAL) and uterocalin. It is a circulatory protein responsible for the transportation of small, hydrophobic molecules, including steroids, free fatty acids, hormones, and prostaglandins [54]. Physiological functions of LCN-2 include the transport of hydrophobic ligands through cell membranes, management of immune system responses, and controlling iron levels [55].

The levels of this protein are low in the healthy human body. However, highly elevated levels were found in various types of cancer, including breast, thyroid, pancreatic, colon, ovarian, and bile duct cancer. Significant levels of LCN-2 have corresponded with high cell growth, increased invasion, and spreading of cancer cells. What is more, LCN-2 enhances the activity of matrix metalloprotease-9, an enzyme, which increases the chance of malignant infiltration and metastasis [56].

LCN-2 is also suspected to be involved in the development of HF and CVD as well as heart hypertrophy. In those conditions, LCN-2 levels are elevated, however, the precise role of LCN-2 is not clear. The enhancement of metalloproteinase activity, leading to matrix degradation, should be taken under consideration as a potential cause [55].

### 4.6. Chemerin

Despite the fact that chemerin, also known as tazarotene-induced gene 2 (TIG2), was discovered in 1997, its role as an adipokine is a relatively new subject. This protein controls adipocyte development through connecting with its receptor (ChemerinR). TIG2 also promotes chemotaxis of dendritic cells and macrophages [55].

High levels of circulating chemerin were identified within several diseases where inflammatory processes are involved in pathogenesis, including metabolic syndrome, obesity and coronary artery disease. Novel works indicate that chemerin serum levels can predict cardiac events in patients with CHF [57] and high chemerin levels are connected with a greater chance of HF occurring [58]. This concludes that the chemerin role in heart diseases should be researched further, and chemerin could be a potential predictor factor for various diseases of the circulatory system.

### 4.7. Retinol-Binding Protein 4

Retinol-binding protein 4 (RBP4) is a 21 kDa protein that works as a vitamin A carrier in a bloodstream. RPR4 as an adipokine is known for enhancing insulin resistance and thus promoting development of obesity [59].

RBP4 is mainly connected with various heart diseases. In CHF, its elevated levels can be used as a prognostic factor [60]. In other conditions, such as hypertension and coronary artery disease, high RBP4 levels can contribute to creation of inflammation and lead to irreversible heart cells damage, resulting in greater chance of development of HF [61].

RPB4 elevated levels in patients with HF can lead to loss control of glycaemia, due to insulin resistance induced by RBP4. However, improving heart work with LV-assisted devices can lead to a decrease in RBP4 levels, resulting in the better control of metabolic function in this patient group [62].

### 4.8. Vaspin

Vaspin is an adipokine secreted mainly by visceral adipose tissue (VAT) and subcutaneous adipose tissue (SAT). It is a member of a serpin superfamily. It is mainly known for being a serine inhibitor with the ability to increase insulin sensitivity, with additional anti-inflammatory effects [63]. In conducted studies, involving patients with metabolic syndrome, vaspin levels were lowered, as well as vaspin’s gene expression [64].

Vaspin’s low levels are strongly connected with the possibility of cardiac events occurring in patients with CHD [55]. Similarly, low levels of vaspin are a remarkable predictor of required hospital treatment due to recurrent acute myocardial infarction (AMI) and HF [65].

Vaspin’s role in heart diseases is still mainly unknown, however, it seems that this adipokine plays a vital role in the development of different cardiovascular diseases. Thus, it needs to be researched further, both as a predictor, and a potential therapeutic target.

### 4.9. Visfatin

Visfatin is an adipokine which was discovered in 2004. As the name suggests, it is mainly produced in visceral adipose tissue (VAT). It is also expressed in human leukocytes, muscles, and hepatocytes [55].

The effects of visfatin are similar to insulin, resulting in lowering glucose levels in blood. Distributions of its function can lead to development of insulin resistance and obesity [66]. As for cardiovascular diseases, visfatin levels were significantly lowered in patients diagnosed with HF, compared to healthy individuals [67]. However, patients suffering from CHD had higher levels of visfatin serum levels and the adipokine was suggested as an independent risk factor of CHD [68]. Those opposite results in two cardiovascular diseases indicate a need for further research for the role of visfatin in CVD.

### 4.10. Omentin-1

Omentin-1 is a newly found, circulating molecule expressed solely by VAT. As established in clinical trials, omentin-1 levels are lowered in plenty of conditions, including insulin resistance, metabolic syndrome, diabetes, cancers, inflammatory diseases, and polycystic ovary syndrome [69].

The role of omentin-1 in cardiovascular diseases is still poorly known. Certainly, decreased serum omentin-1 levels correlate with worse cardiac outcomes in HF patients [70]. Studies conducted on mice with an overexpression of omentin-1 revealed that the adipokine reduces myocardial ischemia-induced HF heart damage by activating mitophagy and preserving mitochondrial homeostasis [71].

### 4.11. Apelin

In 1998, a novel adipokine was discovered—apelin. It binds to the previously discovered receptor APJ, which was “an orphan” until then. When the receptor is activated, it can lead to various effects, including neoangiogenesis, the regulation of the constriction and dilation of blood vessels and enhancement of the heart muscle’s contractility [72].

Apelin effect on CVD is being widely researched. It was found that apelin distributed in humans (as well as APJ agonists) has an inotropic and vasodillatory effect with minimal side effects [73,74]. What is more, patients with diagnosed HF were identified with lower APJ expression, thus limiting the inotropic functions of apelin. The study suggests that exogenous apelin administration could be beneficial, however, the most important question is if it is possible to restore the APJ level and function [75].

All these studies present the possibility of the great role of apelin in the development of CVD, and further research is needed to conclude if the usage of apelin in the treatment of these conditions could be beneficial for patients.

### 4.12. Novel Adipokines: FGF-21 and Neuregulin-4

Fibroblast Growth Factor (FGF)-21 and Neuregulin-4 (Nrg4) are relatively “fresh” additions into the adipokines family. With most of their functions remaining undefined, loss of body weight, loss of adipose tissue, and improved glucose tolerance are some functions that scientists know for certain [15].

FGF21 has emerged as a potential biomarker and therapeutic target in heart failure (HF). Elevated serum levels of FGF21 have been observed in patients with both reduced (HFrEF) and preserved ejection fraction (HFpEF), suggesting its diagnostic value and potential to reflect diastolic dysfunction [76,77]. For in vitro studies, FGF21 exerts cardioprotective effects by mitigating cardiac remodeling processes such as hypertrophy, fibrosis, and inflammation—key contributors to HF progression [78]. Preclinical studies demonstrate that FGF21 can modulate SIRT1 (the protein that plays a role in regulating metabolism, responding to oxidative stress, and modulating inflammation) activity, reduce oxidative stress, and prevent lipid accumulation in cardiomyocytes. Although clinical evidence remains limited, preliminary trials indicate that FGF21 gene therapy and pharmacologically induced FGF21 expression may enhance cardiac function in HF models [79].

Moreover, FGF21 is increasingly recognized as a promising biomarker for predicting the onset and progression of type 2 diabetes mellitus (T2DM). Numerous studies and meta-analyses have shown that patients with T2DM exhibit significantly higher serum FGF21 levels compared to healthy controls [80]. Elevated FGF21 concentrations are also associated with an increased risk of glycemic deterioration and may serve as an alternative to the oral glucose tolerance test for early diabetes prediction. Moreover, the FGF21/adiponectin ratio has emerged as a valuable indicator of worsening glycemic control [81]. Importantly, FGF21 is not only a marker but also plays a protective role in T2DM by enhancing glucose homeostasis, promoting insulin secretion via the PI3K/Akt pathway, and preserving β-cell function through mechanisms such as lipid regulation and autophagy induction (Figure 3).

Nrg4 is secreted solely by brown adipose tissue. What is more, exposure to cold temperatures increases levels of this adipokine in blood [82]. Nrg4 exhibits protective properties against the development of obesity, with its expression in adipose tissue significantly reduced in both obese humans and mice. This decrease is associated with the chronic inflammation characteristic of obesity, as demonstrated by in vitro studies showing that pro-inflammatory cytokines such as TNF-α and IL-1β suppress Nrg4 expression [82]. The overexpression of Nrg4 in mice enhances energy expenditure, increases substrate oxidation, lowers leptin levels, and normalizes the adipokine profile—including adiponectin and adipsin—thereby improving metabolic function [83]. Additionally, Nrg4 promotes angiogenesis in adipose tissue, and its deficiency leads to reduced vascularization and the development of obesity even under normocaloric conditions. Clinically, lower circulating levels of Nrg4 are negatively correlated with body mass index (BMI), waist circumference, and the presence of metabolic syndrome, supporting its potential role as both a biomarker and therapeutic target in obesity treatment [84].

## 5. Flozins and Their Influence on Adipokines

**Figure 5:**
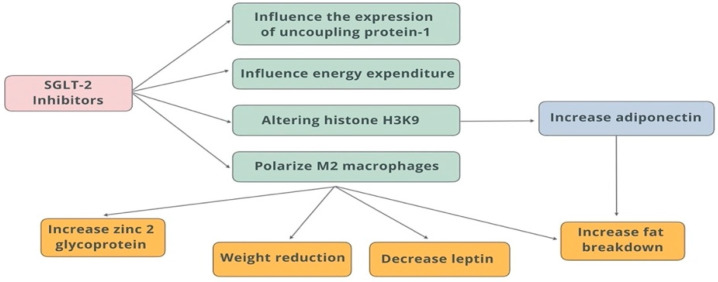
Effects of SGLT-2 inhibitors on adipose tissue.

Flozins (SGLT-2 inhibitors) significantly affect the adipokine profile—in particular, increasing adiponectin levels, which translates into improved insulin sensitivity, reduction in inflammation, and ultimately influencing cardioprotective benefits. This is one of the more important mechanisms for the “add-on” benefits of flozins, beyond just glycemic control, especially in overweight/obese and type 2 diabetic patients. In adipose tissue, M2 macrophages (immune cells that are involved in tissue repair and anti-inflammatory responses) play a role in regulating adipocyte metabolism by promoting adiponectin secretion and reducing inflammation. Alterations in histone H3K9 modifications in adipocytes can affect adipocyte metabolism and contribute to the development of metabolic disorders. The zinc glycoprotein found in adipose tissue regulates insulin sensitivity and glucose metabolism. Its levels are reduced in obesity and type 2 diabetes. Adiponectin and M2 macrophages play an important role in promoting fat breakdown and reducing inflammation in adipose tissue [85,86] (Figure 5).

In animal studies, it was shown that in obese rats with diabetes by altering histone H3K9, dapagliflozin increases the synthesis of 3-hydroxybutyrate, which in turn upregulates adiponectin in adipocytes and increases insulin sensitivity in them. SGLT-2 inhibition increases circulating levels and expression of the zinc glycoprotein 2 gene in type 2 diabetes patients, improving insulin sensitivity [87].

A meta-analysis by Peili Wu et al. [88] assessed the association between SGLT-2 inhibitor treatment and circulating leptin or adiponectin levels in patients with T2DM.

The evaluation involved patients with type 2 diabetes, stratified by age, BMI, and treatment duration. The mean age of the cases studied was 57.8 years, the mean HbA1c level was 64.92 mmol/mol, and the mean duration of the intervention was 20.2 weeks.

The following flozins were used in the study: luseogliflozin, tofogliflozin, dapagliflozin, ipragliflozin). They also noted previous studies conducted on this topic, however, whose results were not consistent, and the relationship between adipokines and SGLT-2 inhibitors remained unclear [89,90,91,92].

In the case of leptin, 6 studies were compared, when pooled in comparison with the placebo group, the use of SGLT-2 inhibitors reduced leptin levels (SMD −0.29, 95% CI −0.56, −0.03; *p* = 0.032) most significantly in the subgroup aged <60 years, with BMI < 30 kg/m^2^ and treatment duration > 24 weeks. For adiponectin, in turn, 10 randomized trials were analyzed. It was shown that treatment with an SGLT-2 inhibitor significantly increased blood levels of adiponectin compared to placebo (SMD 0.30, 95% CI 0.22, 0.38; *p* < 0.001). A statistically significant increase was calculated in a similar age subgroup and body weight to the results of the analysis of leptin concentrations, with no significant association with duration of therapy. In this study, the correlation of adipokines (a decrease in leptin concentrations and an increase in adiponectin concentrations) during flozins therapy suggests an increase in insulin sensitivity, with consequent effects on weight reduction and overall improvement in metabolic homeostasis [88].

Type 2 diabetes mellitus is one of the most significant risk factors for cardiovascular disease representing the leading cause of death in patients with diabetes [93]. In the cardioprotective aspect—adiponectin protects against atherosclerosis through mechanisms at the cellular level described in the section above, resulting in inhibition of the expression of monocyte adhesion molecules and inhibition of vascular smooth muscle cell proliferation [33,34,35].

Zhang H. et al. [94] in their meta-analysis showed that higher levels of adiponectin are associated with a lower risk of coronary heart disease presented in prospective studies. The protective effect of adiponectin was observed in both men and women in the middle-aged population. In the older population, the association was not as statistically significant, due to additional confounding factors such as often additional comorbidities in the older population (i.e., hypertension, diabetes, dyslipidemia and associated additional pharmacotherapy). It was also highlighted that the importance of the possible phenomenon of adiponectin resistance, and the condition of subclinical cardiovascular disease when adiponectin levels may be increased in response to inflammation, which triggers increased expression, synthesis and release—a physiological attempt to limit further endothelial damage [95].

Animal studies have also shown a link between adiponectin and the inhibition of atherosclerosis. Mice with the adiponectin gene turned off showed increased neointima proliferation in response to vascular injury, and in turn, overexpression of the globulin portion of adiponectin in a proatherogenic mouse model reduced the development of atherosclerosis [96,97].

Garvey W.T. et al. evaluated the correlation between the change in leptin and adiponectin levels measured in a randomly selected subgroup of patients with type 2 diabetes receiving canagliflozin 300 mg (n = 100) or glimepiride (n = 100). They showed that canagliflozin increased serum adiponectin levels by 17% compared with glimepiride in patients with type 2 diabetes [98].

Diwan et al. showed adiponectin levels were significantly higher in diabetics than in nondiabetic participants irrespective of gender (*p* ≤ 0.04 in males, *p* ≤ 0.02 in females). Leptin levels were significantly higher in diabetics compared to nondiabetics (*p* ≤ 0.001) in both males and females [99].

Hanson et al. demonstrated a significant and substantial reduction in body weight, and thus a decrease in adipose tissue mass, with a consistent reduction in serum leptin concentrations, despite the maintenance of 24-h energy expenditure and self-reported appetite. They report that this is the first study of its kind to determine the effect of dapagliflozin over 12 months of therapy on characteristics of metabolic and appetite responses in combination with dietary carbohydrate restriction in patients with type 2 diabetes and class 3 obesity. They demonstrated a significantly greater reduction in serum leptin concentrations in response to dapagliflozin therapy [100].

Animal studies have also confirmed the beneficial effects of adiponectin on the metabolic profile. Fruebis et al. showed adiponectin treatment supported muscle insulin sensitivity in mice, which was associated with a reduction in body weight [101]. Nishitani et al. identified the effect of dapagliflozin in KKAy mice (KKAy mice treated with dapagliflozin (KKAy  +  Dapa). They found that dapagliflozin tended to increase plasma adiponectin levels and upregulate adiponectin expression in adipose tissue compared with diabetic KKAy mice non treatment dapagliflozin [87].

## 6. Metabolic Effects of SGLT-2 Inhibitors in Adipose Tissue and Influence on Hormones Secreted by the Tissue

Adipose tissue is a metabolically active organ that responds to a variety of hormonal, inflammatory and metabolic interactions with other organs. There are many individual factors such as genetic factors (e.g., gene polymorphisms) physical activity, adipose tissue distribution, adipocytokine receptor sensitivity, insulin resistance that influence the complex interaction between adipose tissue and the cardiovascular system [102,103,104]. Adipose tissue may mediate hemodynamic signals and potentially influence the development of heart failure [105]. The increased volatilization of free fatty acids results in increased myocardial oxygen consumption, accumulation of toxic lipid metabolites, and generation of reactive oxygen species. In addition, insulin resistance leads to hyperinsulinemia and chronic systemic hyperglycemia, which results in cardiomyocyte damage caused by hyperglycemia or glucotoxicity. A state of chronically elevated insulin levels is a factor leading to hyperleptinemia. Intramuscular fat accumulation may contribute to cardiomyocyte injury through oxidative and non-oxidative [106,107].

Since hyperglycemia is directly related to oxidative stress, the normoglycemic effect of SGLT-2 inhibitors is thought to be an indirect antioxidant mechanism that further reduces free radical production [108]. The main mechanisms of SGLT-2 inhibitors in cardioprotection improve cardiac cell metabolism, abolish ventricular stress, inhibit Na^+^/H^+^ exchange in myocardial cells and reduce cardiac cell necrosis and myocardial fibrosis [109,110]. The pleiotropic effects of flozins include blood glucose-dependent and independent mechanisms. A review by Bodnar et al. outlines the antioxidant effects of these drugs, confirming the effects on reducing pro-inflammatory cytokine levels in various animal experiments in available studies [111].

Another explanation for the beneficial effects of flozins on inflammation and oxidative stress may be the effect on adipokine profile and leptin [112,113]. Adipokines secreted from adipose tissue have been shown to contribute to the remodeling associated with obesity/metabolic syndrome and cardiovascular disease [55,114]. SGLT-2 inhibitors have been found to reduce epicardial adipose tissue and leptin levels, which was associated with reduced inflammation and oxidative stress [98,115,116]

Leptin may increase the risk of consequences induced by oxidative stress in the heart through elevated NHE1 (Na^+^/H^+^ exchanger-1) activity. It has been shown that flozins inhibit NHE1 and thus protect the heart against the effects of oxidative damage, leading to, for example, the development of HF. Additionally, the decreased leptin levels observed with SGLT-2 inhibition may be due to a reduction in adipose tissue [117].

## 7. Pleiotropic Effects of SGLT-2 Inhibitors in the Aspect of Clinical Trials

SGLT-2 inhibitors are able to counteract oxidative damage and protect tissues from the destructive effects of free radicals, not only through their glucose-lowering action but also through the supportive action of the antioxidant system. Flozins contribute to the improvement of the redox state [118]. The effect of SGLT-2 inhibitors on reducing oxidative stress may be reflected in the reported clinical benefits observed in cardiovascular and renal indices reported in the EMPA-REG and CANVAS clinical trials [119,120,121]. Inflammation, oxidative stress, and glycaemic fluctuations lead to atrial fibrosis and dilation with increased electromechanical delay and increased vulnerability to atrial arrythmias. In a new report, Mariani et al. demonstrated that SGLT-2 inhibitors therapy, by restoring calcium and sodium homeostasis, exerting antioxidative and anti-inflammatory effects, and reversing cardiac remodeling, confers a beneficial antiarrhythmic impact. They showed that in patients with HFrEF who had an ICD or CRT-D implanted, the number of arrhythmic episodes decreased by 73.8% within one year of initiating flozin therapy. The largest reduction was noted in episodes of atrial fibrillation (AF), followed by non-sustained ventricular tachycardia (NSVT) and sustained ventricular tachycardia (SVT) [122]. Epidemiological studies have identified a relationship between obesity and HFpEF [123].

Recent studies using flozins show that they reduced the percentage of unfavorable endpoints in HFrEF and also in HFpEF. Although adipokines were not measured in these studies, their results document varying efficacy depending on BMI. Dapagliflozin reduced the risk of worsening heart failure or cardiovascular death in HFpEF and HFrEF in comparison to placebo in patient with BMI > 30 kg/m^2^. On the contrary, empagliflozin reduced the combined risk of cardiovascular death or hospitalization for heart failure in patients with HFpEF and BMI < 30 kg/m^2^ reported in the DELIVER and EMPEROR-Preserved trials [124,125].

## 8. Conclusions

Adipose tissue plays a multifaceted role—from energy storage to regulation of thermogenesis, to numerous endocrine and immune functions. The adipokines it secretes are key regulators of metabolism and inflammation. Abnormalities in adipokine production (e.g., a decrease in adiponectin and an increase in leptin or pro-inflammatory cytokines) contribute to the development and progression of obesity, insulin resistance, type 2 diabetes, and cardiovascular complications. Recent research has placed increasing importance on identifying mechanisms regulating adipose tissue function and adipokine secretion as potential therapeutic targets in metabolic diseases (although adiponectin remains the best-studied and described adipokine).

Clinical and observational studies suggest that flozin therapy may lead to an increase in serum adiponectin levels. The mechanism is not fully understood, but it is presumed that improved insulin sensitivity, reduced oxidative stress, and some reduction in fat mass (especially visceral fat) may contribute to increased adiponectin secretion. A higher level of adiponectin is associated with improved insulin sensitivity and anti-inflammatory effects. Flozins can enhance this effect by lowering blood glucose and reducing chronic inflammation in obesity. Adiponectin influences lipid metabolism, and drugs in the flozin group may further promote triglyceride reduction and favorably modulate HDL levels, indirectly implicating their cardioprotective effects. Increasing adiponectin levels can support long-term improvement of metabolic control. The antihyperglycemic, antiatherosclerotic, and anti-inflammatory properties of adiponectin make it an attractive therapeutic target for the treatment of obesity and insulin resistance. However, the difficulty of developing therapies targeting adiponectin function remains hampered by its complex structure and regulation as described in this review.

We find that the number of studies on specific types of SGLT-2 inhibitors as well as individual adipokines is relatively small, so it is difficult to study the effects of each type of SGLT-2 inhibitor on specific adipokines. It is uncertain whether the changes in adipokines are a direct effect of SGLT-2 inhibitors, or secondary to reduced adipose tissue or regulation of adipose tissue function. Therefore, further studies are needed in the future to clarify the relationship between the use of specific SGLT-2 inhibitors and adipokine changes.
